# Early plaque formation on PTFE membranes with expanded or dense surface structures applied in the oral cavity of human volunteers

**DOI:** 10.1002/cre2.344

**Published:** 2020-11-09

**Authors:** Alberto Turri, Emina Čirgić, Furqan A. Shah, Maria Hoffman, Omar Omar, Christer Dahlin, Margarita Trobos

**Affiliations:** ^1^ Department of Biomaterials, Institute of Clinical Sciences, Sahlgrenska Academy University of Gothenburg Gothenburg Sweden; ^2^ The Brånemark Clinic, Public Dental Service Region Västra Götaland Gothenburg Sweden; ^3^ Department of Orthodontics University Clinics of Odontology, Public Dental Service, Region Västra Götaland Gothenburg Sweden; ^4^ Department of Orthodontics, Institute of Odontology, Sahlgrenska Academy University of Gothenburg Gothenburg Sweden; ^5^ Vice Deanship for Postgraduate Studies and Scientific Research, College of Dentistry Imam Abdulrahman bin Faisal University Dammam Saudi Arabia; ^6^ Department of Oral, Maxillofacial Surgery and Research and Development NU‐Hospital Organisation Trollhättan Sweden

**Keywords:** bacteria, biofilm, GBR, human model, PTFE membrane, randomized, staphylococci

## Abstract

**Objectives:**

This clinical randomized study aimed to evaluate the early plaque formation on nonresorbable polytetrafluoroethylene (PTFE) membranes having either a dense (d‐PTFE) or an expanded (e‐PTFE) microstructure and exposed to the oral cavity.

**Material and Methods:**

Twelve individuals were enrolled in this study. In a split‐mouth design, five test membranes (e‐PTFE) with a dual‐layer configuration and five control membranes (d‐PTFE) were bonded on the buccal surfaces of posterior teeth of each subject. All study subjects refrained from toothbrushing during the study period. Specimens were detached from the teeth at 4 and 24 hr and subjected to viability counting, confocal microscopy, and scanning electron microscopy. Plaque samples were harvested from neighboring teeth at baseline, 4, and 24 hr, as control. Wilcoxon signed rank test was applied.

**Results:**

No bond failure of the membranes was reported. Between the early and late time points, viable bacterial counts increased on all membranes, with no difference between the test and control. The number of *Staphylococcus* spp. decreased on the tooth surfaces and increased on both membranes overtime, with a significant difference compared to teeth. The total biomass and average biofilm thickness of live and dead cells were significantly greater at the d‐PTFE barriers after 4 hr.

**Conclusion:**

This study demonstrated that the e‐PTFE membrane was associated with a lesser degree of biofilm accumulation during the initial exposure compared to the d‐PTFE membrane. The present experimental setup provides a valuable toolbox to study the *in vivo* behavior of different membranes used in guided bone regeneration (GBR).

## 1 INTRODUCTION

The development of barrier membranes for the regeneration of bone deficiencies has been of utmost importance in implant dentistry in the past 30 years. Since then, a large variety of membranes have been introduced (Omar, Elgali, Dahlin, & Thomsen, 2019; Sanz et al., 2019). Nowadays, absorbable membranes are often selected for guided bone regeneration (GBR) procedures mainly because of their clinical manageability and the successful outcome in combination with different grafting materials, but they are not the first choice to keep appropriate space unless bone defect morphology is favorable (Benic & Hammerle, 2014). Thereby, in many situations with disadvantageous bone deficiencies, nonresorbable polytetrafluoroethylene (PTFE) membranes are used. Despite their effectiveness and predictability (Jepsen et al., 2019), there are still complications such as soft‐tissue dehiscence and membrane exposure which could jeopardize the clinical outcome, as recently described in a systematic review (Garcia et al., 2018). The latter review revealed that the sites without membrane exposure achieved 74% more horizontal bone gain than those with exposure. Data from Lim and coworkers (Lim, Lin, Monje, Chan, & Wang, 2018) reported a 16.8% soft‐tissue complication rate (i.e., soft‐tissue dehiscence, acute infection/abscess, membrane exposure) when using either nonresorbable or resorbable membranes. Apart from these clinical outcomes, the early processes associated with membrane exposure, including membrane vulnerability to bacterial accumulation and biofilm formation, remains largely unknown.

Reduction of the number of bacteria colonizing the membranes can lead to more favorable healing and successful regeneration (Yaghobee, Samadi, Khorsand, Ghahroudi, & Kadkhodazadeh, 2014; Yoshinari et al., 1998). However, the literature on barrier membranes and their potential antimicrobial behavior during GBR is still very scarce. For instance, it has been suggested that dense PTFE (d‐PTFE) is more resistant to bacterial penetration since the pore size is less than 0.3 𝜇m (Bartee & Carr, 1995). In a recent *in vitro* investigation (Trobos, Juhlin, et al., 2018), a new generation of expanded PTFE (e‐PTFE) membrane with a dual‐layer configuration was evaluated. It was demonstrated that the dual‐layered e‐PTFE membrane withstands bacterial permeability while associated with less colonization and biofilm formation by *Streptococcus oralis* compared to a d‐PTFE membrane.

The objective of this clinical study was to evaluate early dental plaque formation on a dense and an expanded PTFE nonresorbable membranes exposed to the oral cavity.

## 2 MATERIALS AND METHODS

Twelve healthy individuals of both genders, aged between 18 and 50 years, and with a minimum of 24 teeth were invited to participate in this study. The exclusion criteria were subjects with periodontal disease or caries lesions, smokers, pregnant or breastfeeding women, subjects that used chemical plaque control agents or antibiotics in the previous 6 months, subjects with dental and/or skeletal deep bite, and subjects with mouth breathing or with parafunctional habits such as bruxism. The evaluation of the medical and dental history of the volunteers was carried out by two examiners (AT, EC) through a clinical examination and an individual case history to identify possible risk factors. All participants were informed about the objectives, methodology, and purpose of the study, and those who agreed to participate were required to provide verbal and written consent prior to entry. This study was reviewed and approved by the Local Ethical Review Board in Gothenburg — Sweden (Dnr 379‐14).

### 2.1 Membrane discs

Round specimens (4 mm diameter and 0.25 mm thickness) were punched from polytetrafluoroethylene (PTFE) membranes under sterile conditions. Test and control membranes were used. The test membrane was the nonresorbable expanded PTFE (e‐PTFE) (NeoGen™, Neoss Ltd; UK) with a dual‐layer configuration, where the layer thickness and the degree and direction of expansion (multidirectional) were optimized to achieve a distinct membrane structure. The control membrane was the commercially available nonresorbable dense polytetrafluoroethylene (d‐PTFE) membrane (Cytoplast™ TXT‐200, Osteogenics Biomedical Inc., TX, USA). Detailed description of the material properties of both membranes can be found at Trobos and coworkers (Trobos, Juhlin, et al., 2018).

### 2.2 Study design

At the start of the study, the participants were asked to refrain from all mechanical and chemical plaque control measures for the next 24 hr. The experimental phase comprised two plaque accumulation periods of 4 and 24 hr.

**FIGURE 1:**
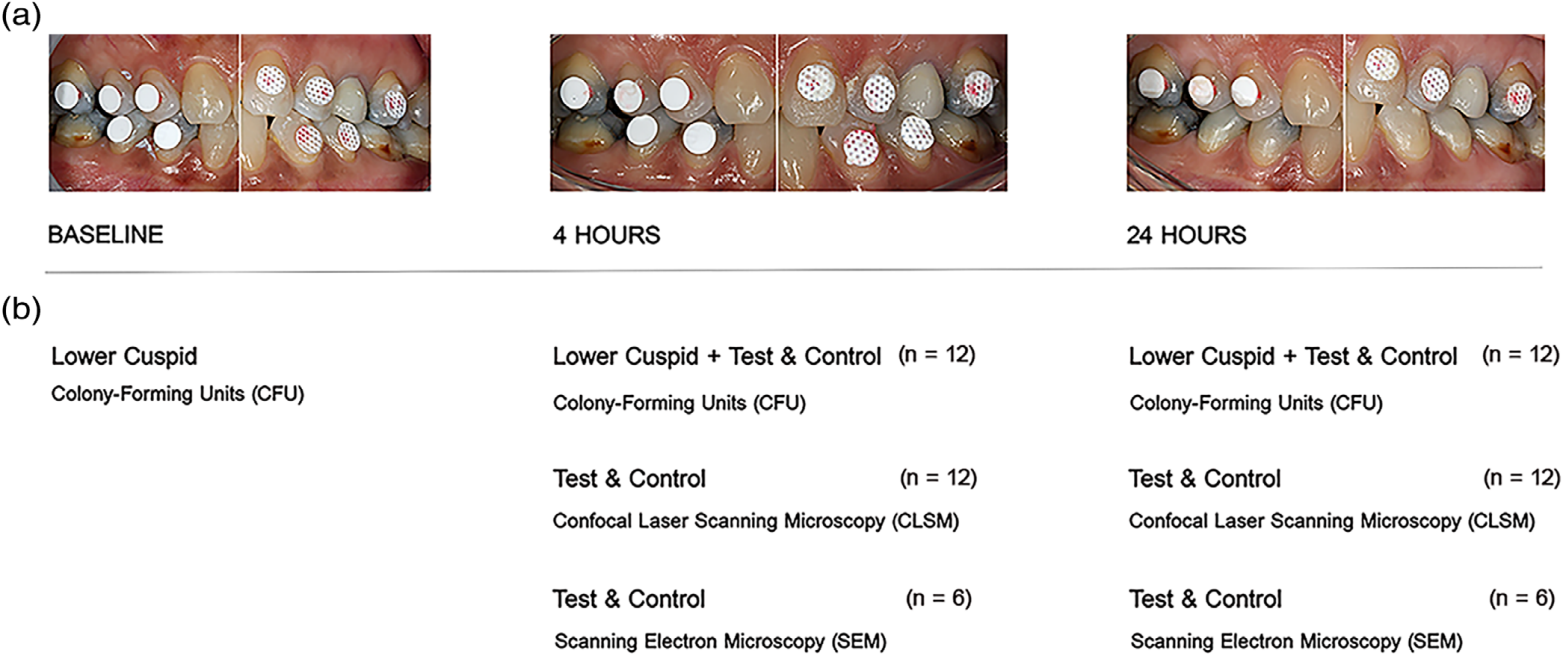
(a,b) Outline of the study: (a) View of the PTFE specimens directly applied onto the teeth surfaces. Test membranes are visible on the left side and the control membranes on the right side (experimental protocol n°1) at baseline, 4 hr, and 24 hr before retrieval. Two protocols were followed where test and control were randomized to left or right side of the mouth; (b) sampling protocol including the retrieved samples and associated analytical techniques at baseline, 4 hr, and 24 hr. One test and one control membrane were retrieved per volunteer and time point for colony‐forming units (*n* = 12) and confocal laser scanning microscopy (*n* = 12). One test and one control membrane were retrieved per volunteer at either 4 hr (*n* = 6) or at 24 hr (*n* = 6) for scanning electron microscopy analysis

Before bonding the membrane discs onto the tooth surfaces and at each time point before membrane retrieval (4 and 24 hr), supragingival plaque samples were harvested with a sterile curette from the buccolingual surfaces of a lower cuspid, which served as control for microbiological analysis in each patient. After that, professional mechanical tooth cleaning was performed with the use of abrasives and rubber cups. The membrane specimens were then fixed on the buccal surfaces of the experimental teeth: premolars and first molars in the upper jaw (six teeth) and second premolars and first molars in the lower jaw (four teeth) (Figure 1a). When conditions such as extensive restorations on the selected teeth or hypodontia were encountered, the membrane specimens were fixed either on other available posterior teeth or on the cuspids.

A total of 10 membrane specimens were bonded on each subject, using a moisture‐tolerant light cure bonding system (SmartBond®, Gestenco International AB, Sweden). The assignment of test and control membranes on the right or left side of the participants was randomized by lottery in a split‐mouth design according to two experimental protocols that were created to allocate equal number of specimens to the respective quantitative (*n* = 12) and qualitative (*n* = 6) analyses (Figure 1b).

**FIGURE 2:**
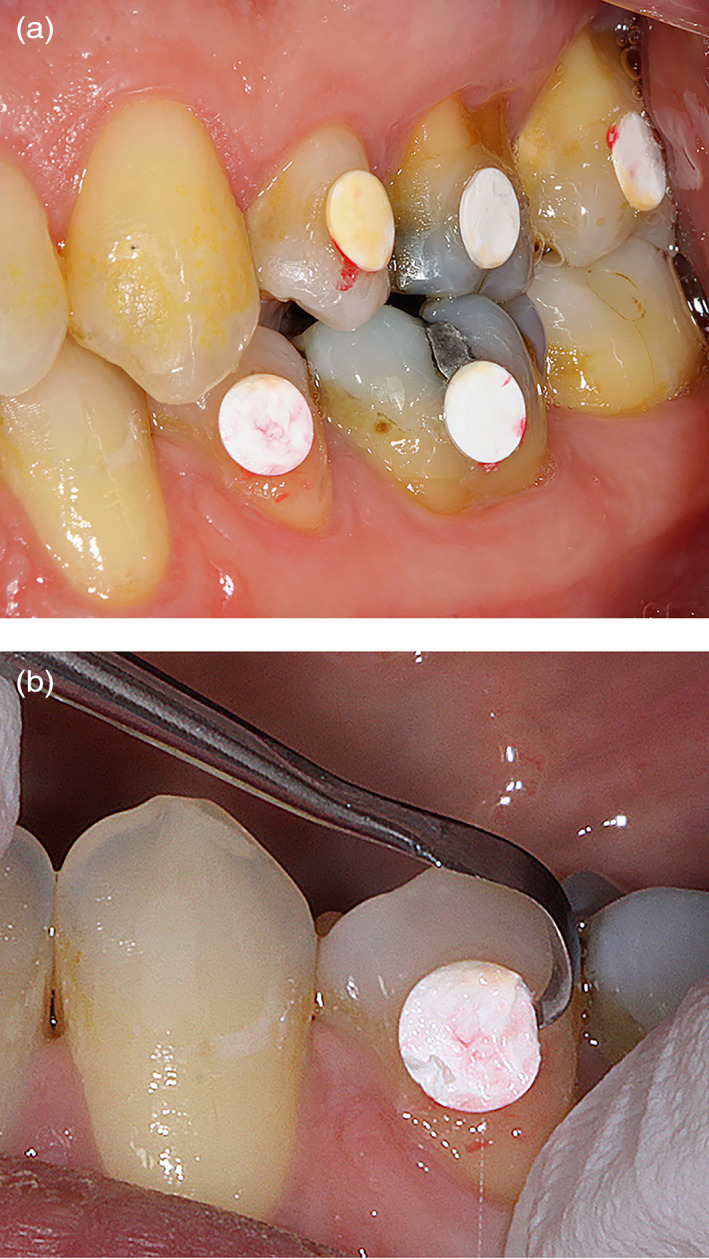
(a,b) Example of the mechanical retrieval of e‐PTFE membrane (test) after 4 hr of plaque accumulation

After 4 hr, all participants were recalled to retrieve the first plaque‐covered specimens with small forceps, for a total of four or six membrane discs, according to the corresponding protocol (Figure 2a,b). At the end of the experimental phase (24 hr), the remaining four or six membrane discs from the upper jaw were retrieved. At the two time points, the specimens were carefully detached from the buccal surfaces of the teeth and transferred to individual tubes and well plates for the respective microbiological analyses (Figure 1b). At the end of the experimental phase, professional mechanical tooth cleaning was performed in the entire dentition with the use of abrasives and rubber cups.

### 2.3 Microbiological sampling and analyses

#### 2.3.1 Viability counting (colony‐forming units—CFU Counting)

CFU counting was performed as described before (Trobos, Johansson, et al., 2018). Briefly, after each time point, each membrane was collected in an ESwab™ (Copan Italia S.p.A., Brescia, Italy) containing 1 ml transport medium and immediately transported to the laboratory to be processed as follows: the ESwab™ tube was vortexed for 30 s at 3200 rpm, sonicated for 5 min at 40 kHz in an ultrasonic bath and vortexed again for 30 s in order to dislodge the dental biofilms from the membranes and break bacterial cell aggregates into single cells. The number of viable colony‐forming units (CFUs) was assessed by quantitative culturing. The sonicated suspension was diluted in a series of four 1:10 dilutions in 0.9% saline. Hundred μl from dilution 10^−4^ and 10^−2^ was then spread on 5% horse Columbia blood agar plates (H207) and brucella agar plates (B350) (Media Department, Clinical Microbiology laboratory, Sahlgrenska University Hospital, Sweden) and incubated at 37°C aerobically for 2 days and anaerobically for 5 days, respectively, before counting. In addition, the presence and number of staphylococci were assessed by spread‐plating dilution 10^−2^ and the undiluted sample on selective Staph‐plates (S316, Media Department) and incubated aerobically at 37°C for 2 days. The experiment was performed on duplicate agar plates. If growth was observed on the Staph‐plates, then one colony was subcultured on the same medium, and the strain was stored in cryotubes containing TSB and glycerol at −80°C. The staphylococcal strains were further identified using CHROMagar™ Staph aureus (CHROMagar, Paris, France).

#### 2.3.2 Confocal scanning laser microscopy (CLSM) analysis of the biofilms

One test and one control membrane per volunteer and time point, containing the accumulated dental biofilm, were carefully glued on the base of 60 mm petri plates using SmartBond®, ensuring that the membranes were placed as flat as possible in the center of two 9 mm diameter 1 mm deep silicone frames Press‐to‐Seal™ (Molecular Probes, Oregon, USA). Immediately thereafter, membranes were covered with 200 μl of staining solution (3:3:1000 of SYTO® 9, propidium iodide (PI) and saline) of FilmTracer™ LIVE/DEAD® Biofilm Viability kit (Molecular Probes) and incubated at RT for 30 min in the dark, in order to stain live (green) and dead (red) cells. Thereafter, stain was removed, and membranes were immersed in saline for *in situ* visualization of biofilms using the C2plus confocal microscopy system (Nikon, Japan) and a Plan 100×/1.10 water dipping objective. The excitation/emission maxima were 488/500 nm for SYTO® 9 and 561/785 nm for PI. For each membrane, five z‐stacks (step size 3 μm) were collected (four in the corners and one in the center). Automatic thresholding was applied to the biofilm images and then processed and quantified using Comstat2 software (Lyngby, Denmark) (Heydorn et al., 2000) in order to determine:

1. Biofilm biomass (μm^3^/μm^2^) which is volume per unit area representing how much of the image stack is covered by bacteria (live and dead);
2. Average thickness of the biofilm (μm) is the average of all set pixels in the 2D image, which informs about the average height of the biofilm, ignoring the presence of pores or voids in the biofilm;
3. Area occupied (%) by the first biofilm layer is the area occupied by biomass in the first stack image;
4. Surface to volume ratio (surface area/bio‐volume, μm^2^/μm^3^) reflects the fraction of the biofilm that is exposed to the nutrient flow.

### 2.4 Scanning electron microscopy (SEM)

At 4 and 24 hr, the respective six membrane samples (for each time point) containing biofilms were carefully rinsed in saline and fixed in modified Karnovsky's fixative for 2 hr at 4°C, rinsed with 0.15 M Na‐cacodylate buffer, stained with 1% osmium tetroxide (OsO_4_) for 2 hr at 4°C, rinsed again with 0.15 M Na‐cacodylate buffer, dehydrated in a graded ethanol series (70, 80, 90, 95, and 100% ethanol, 5 min each step) and allowed to air‐dry overnight. The samples were then mounted on aluminum stubs using conductive silver paint and Au sputter‐coated (~15 nm) for scanning electron microscopy (SEM) (Ultra 55 FEG SEM, Leo Electron Microscopy Ltd, UK) operated in the secondary electron mode at 5 kV accelerating voltage and ~5 mm working distance.

### 2.5 Statistics

The comparisons between test and control membranes, as well as between test or control membranes versus teeth, were statistically evaluated as nonparametric paired samples where Wilcoxon matched‐pairs signed rank test was used. Friedman test was applied to compare time‐points within the three different groups (baseline, 4 hr, 24 hr). Mean values ± *SEM* are provided, and *p‐value < .05* was used as the level of significance. Dot plots were produced to graphically represent the frequency distribution of the patient population with regard to the colony‐forming unit (CFU) counting of staphylococci at baseline, 4 and 24 hr. Statistical analyses were performed with GraphPad Prism version 8.1.0 for macOS (GraphPad Software, La Jolla, California, USA).

## 3 RESULTS

All 120 applied membranes on the tooth surfaces of the 12 volunteers were available for analyses after the respective biofilm accumulation periods.

**FIGURE 3:**
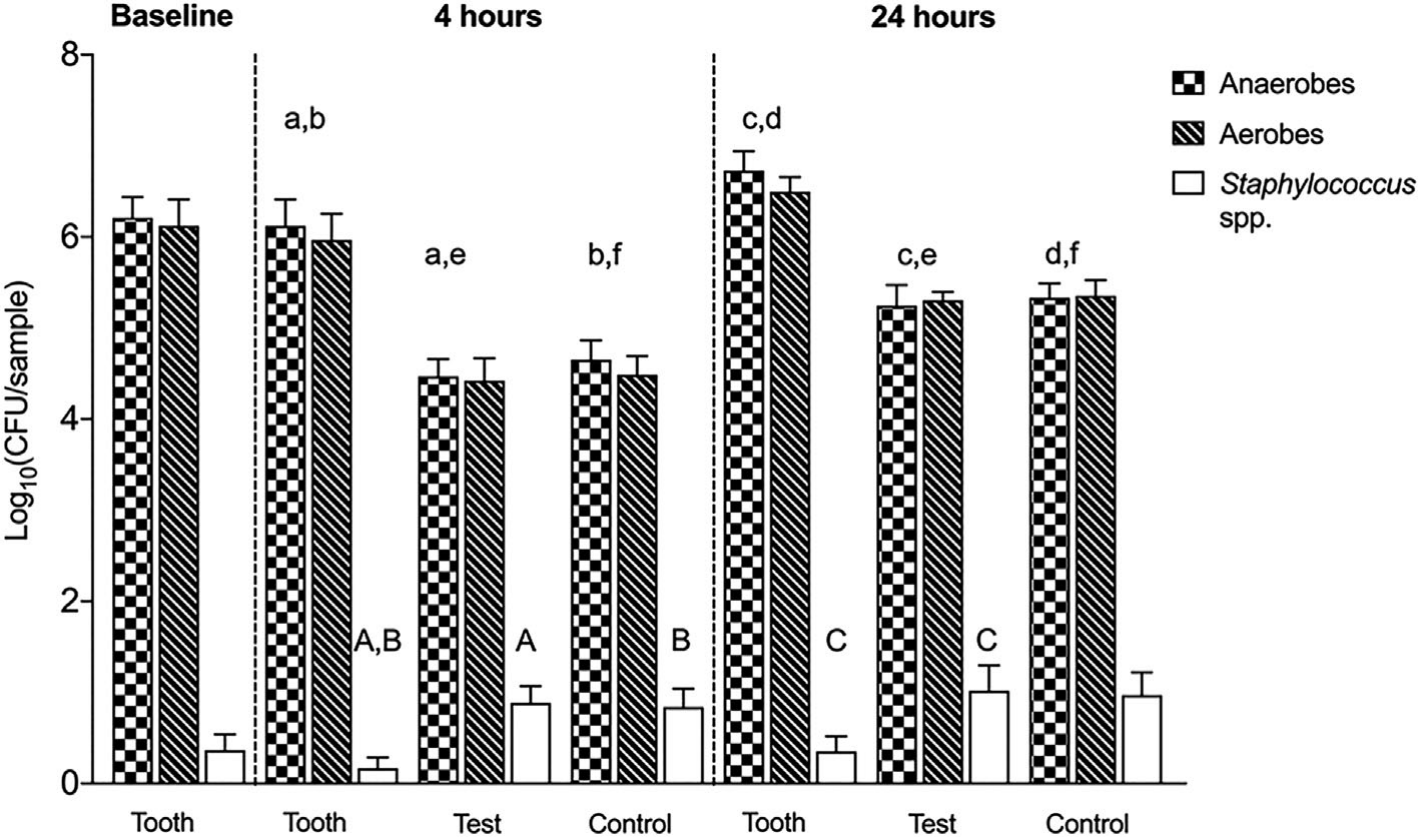
Biofilm formation on natural dentition (tooth), e‐PTFE (test), and d‐PTFE (control) membranes at baseline and after 4 and 24 hr as measured by colony‐forming unit (CFU) counting. Data represent mean ± *SEM*. Bars that share the same letters are significantly different (*p* < .05)

**FIGURE 4:**
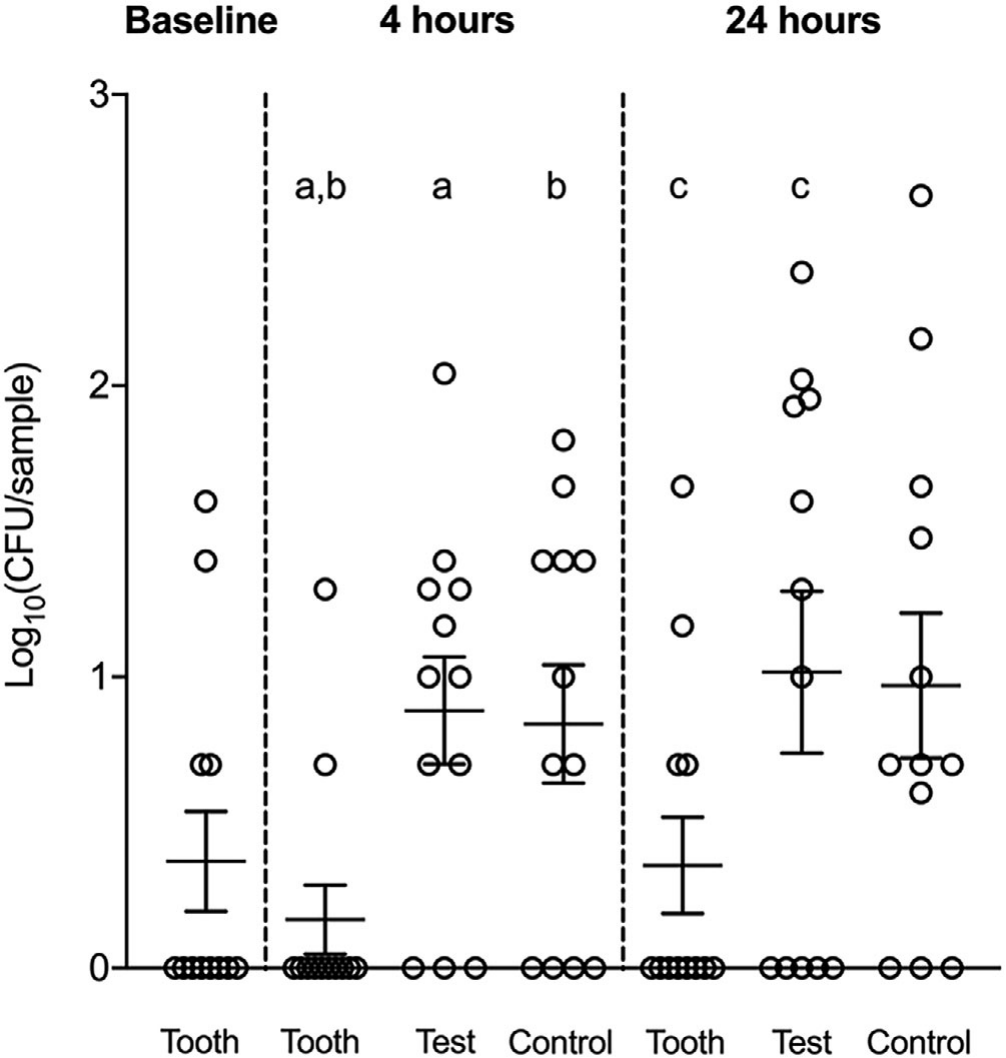
Colonization of *Staphylococcus* spp. on natural dentition (tooth), e‐PTFE (test), and d‐PTFE (control) membranes at baseline and after 4 and 24 hr as measured by colony‐forming unit (CFU) counting. Data represent mean ± *SEM*. Dot plots that share the same letters are significantly different (*p* < .05)

Between the early (4 hr) and late (24 hr) time points, viable CFU counts increased on all membranes, with no difference between test and control (Figure 3). The number of viable aerobes and anaerobes was greater on the tooth surfaces compared to either type of the membranes at both time points. On the other hand, the number of staphylococci had a different pattern of growth overtime, decreased on the tooth surface and increased on both membranes, with a significant difference between tooth surface and both membranes at 4 hr and the test membrane at 24 hr (Figure 4). All saved staphylococcal strains (except from one volunteer) were identified as *Staphylococcus aureus*.

**FIGURE 5:**
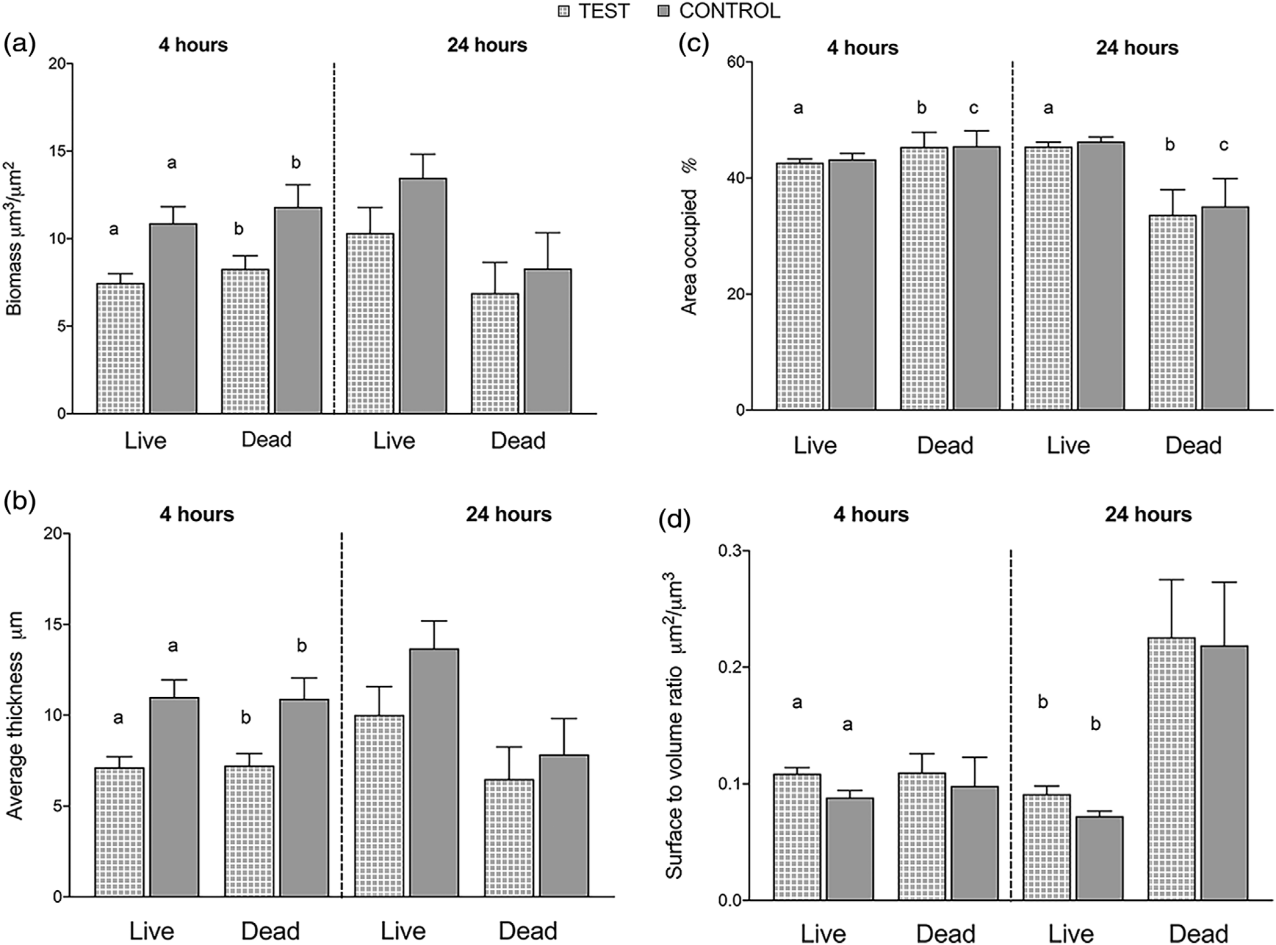
(a–d) Quantification of biofilm formation by confocal scanning laser microscopy (CLSM). 3D images were analyzed by Comstat2 software, and the following parameters were analyzed: Biofilm biomass (μm^3^/μm^2^) (a), average thickness of biofilm (μm) (b), area occupied by the first biofilm layer (%) (c), and surface to volume ratio (μm^2^/μm^3^) (d). Data represent mean ± *SEM*. Bars that share the same letters are significantly different (*p* < .05)

Quantitative analysis of biofilm CLSM images showed an increase in biomass of live cells and a reduction of dead biomass between 4 and 24 hr for both test and control membranes (Figure 5a). The amounts of both live and dead biomass were greater at 4 hr on the control than on the test membrane, while no significant difference could be observed between the two membranes at 24 hr. The biofilm average thickness of either live or dead biomass was also significantly greater on the control membrane compared to test at 4 hr but not at 24 hr (Figure 5b). The area occupied by live and dead bacteria at the first biofilm layer did not reveal any difference between test and control membranes (Figure 5c). Between the early (4 hr) and late (24 hr) time points, the area occupied by live cells increased on the test membrane and the area occupied by dead cells decreased on both membrane types. The surface area covered by live bacteria was greater on the test membrane than on the control at both time points, while no differences between the membranes could be observed for the surface area covered by dead biomass, despite a general increase from 4 to 24 hr on both test and control membranes (Figure 5d).

**FIGURE 6:**
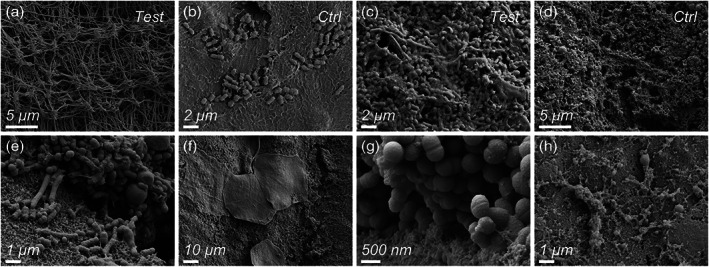
(a–h) Scanning electron microscopy images providing qualitative evaluation of dental plaque formation on PTFE membranes, test (a,c) and control (b,d) at 4 hr (a,b) and 24 hr (c,d). At 4 hr, oral bacteria colonized the membrane surface in sparsely populated monolayers, leaving large areas of the membrane surface visible (a,b). At 24 hr, membranes were densely colonized by oral bacteria in biofilms (c,d). Different bacterial cell morphologies were observed (e), as well as clusters of oral mucosal squamous epithelial cells (f). At 24 hr, several micrometers thick biofilms were found (g). The extracellular polymeric substance (EPS) was observed at 4 and 24 hr (h)

Based on qualitative SEM analysis, at 4 hr, oral bacteria colonized the membrane surface in sparsely populated monolayers, leaving large areas of the membrane surface visible (Figure 6a,b). In comparison, at 24 hr, the membrane surface was densely colonized by oral bacteria in multilayered arrangements (Figure 6c,d). Infrequently, the membrane material surface could be seen, probably attributable to artifacts arising from handling and sample preparation (Figure 6a,b). The multispecies character of the biofilms was evident. The most prevalent bacterial cell morphology was cocci, ranging in size from 300 nm to ≈1.5 μm diameter, followed by bacilli and coccobacilli (Figure 6e). Clusters of oral mucosal squamous epithelial cells were also seen on the membranes and were intermixed with bacterial cells (Figure 6f). At 24 hr, the membrane surface and the biofilm were frequently separated by a structureless, bacteria‐free layer, which was several micrometers in thickness, presumably food debris (Figure 6g). The extracellular polymeric substance (EPS) also differed in the overall appearance between 4 and 24 hr (Figure 6h). While at 4 hr, the EPS exhibited a discrete/particulate character embedding relatively few bacterial cells, the EPS at 24 hr appeared less discrete and more finely fibrillar. In areas where the membrane surface was visible, no visual evidence of membrane damage due to the bonding system was observed, except for traces left by mechanical manipulation during retrieval of the membrane from the tooth surface and sample preparation.

## 4 DISCUSSION

A deeper insight on the microbial communities at their natural habitats is critical to improve our knowledge on the role that bacterial colonization plays in the regenerative procedures. In the present study, a novel combination of a clinical model for dental biofilm formation, together with different analytical procedures for bacterial biofilm characterization, is introduced. The model and the experimental setup were employed to investigate the *in vivo* microbiological behavior on barrier membranes when exposed to plaque formation in the oral cavity.

In the present study, it was demonstrated that the two types of nonresorbable membranes were colonized by a diverse bacterial flora. The present new generation of e‐PTFE is made up of two homogenous layers where the inner layer is characterized by a more multidirectional orientation of the PTFE fibrils compared to the outer layer. Its topography consists of an open microstructure of the inner layer of the membrane, intended to face the bone compartment, that would favor the regeneration capacity by increasing the areas of bone ingrowth into the fibrils. On the other hand, the semiopen structure of the outer layer would allow the adhesion of connective tissue cells and fibers thus promoting wound stability and soft‐tissue integration. Due to its semiopen structure, the outer surface of the e‐PTFE membrane may theoretically become a potential trap for bacteria during intraoral manipulation and/or after membrane exposure. The surface topography and roughness are recognized to be of utmost importance in the extent of adhesion that is essential for a successful biofilm growth phase (Frojd et al., 2011). This is supported by different morphological studies on plaque growth, in which bacteria started to accumulate in microretentive areas of the substratum surfaces (Lie, 1979; Teughels, Van Assche, Sliepen, & Quirynen, 2006; Zucchelli, Cesari, Clauser, & DeSanctis, 1998). Results from the viability counting disclosed that similar numbers of viable aerobes and anaerobes colonized the outer surfaces of both e‐PTFE and d‐PTFE membranes at 4 and 24 hr, which was further confirmed visually by SEM. Different topographical features such as the micron‐range fibril net structure on the e‐PTFE and the macrosurface indentations on the d‐PTFE would represent a good substrate for bacterial adhesion and may have equally contributed to the plaque formation.

In the oral cavity, the initial bioadhesion of microorganisms may not only depend on the topographical characteristics of the substrate but also on the protein adsorption on the surfaces (pellicle) and surface free energy (Teughels et al., 2006). Generally, hydrophobic surfaces, such as PTFE, are more protein adsorbent than hydrophilic surfaces (Müller, Lüders, Hoth‐Hannig, Hannig, & Ziegler, 2010). Moreover, previous studies revealed a positive correlation between biofilm formation and surface free energy of different tested materials (Koseki et al., 2014; Pereni, Zhao, Liu, & Abel, 2006). The material characterization of the present membranes had been previously described (Trobos, Juhlin, et al., 2018) where both the e‐PTFE and d‐PTFE membranes revealed comparable surface free energy (10.83 and 12.9 mN/m, respectively, for the outer layer of e‐PTFE and d‐PTFE). This could, at least partly, explain the comparable plaque‐retaining capacity with regard to cell viability.

In the present study, the pattern of bacterial colonization and the 3D biofilm structure were found to be different between expanded and dense PTFE membranes. Visualization and quantification of live and dead cells at different stages of biofilm development revealed a distinct mode of action of the two PTFE membranes in regard to biofilm accumulation. At the two time points, the area of the substratum colonized by the first layer of bacterial cells (area occupied in %) did not differ between test and control membranes meaning that the first bacterial cells adhered to both membranes in a similar manner finding new empty areas to attach. However, the development and 3D structure of biofilms after the initial adhesion was markedly different between e‐ and d‐PTFE, as measured by the other CLSM parameters, especially at the early time point (4 hr). The total biofilm biomass and average thickness of the live and dead cell populations were significantly greater on d‐PTFE membrane after 4 hr exposure to the oral cavity. Despite not statistically significant, a similar trend was observed after 24 hr.

From a clinical point of view, d‐PTFE membranes are produced with a low porosity (<0.3 𝜇m) in order to protect the grafting material and the initial healing clot from bacterial contamination (Carbonell et al., 2014). The present study indicates that the expanded (multifibrillar) structure of nonresorbable PTFE membranes may not be the condition favoring the adhesion and proliferation of bacteria, since it showed thinner biofilm and less live and dead biomass than the d‐PTFE. This observation, together with the recently demonstrated efficacy against bacterial penetration (*S. oralis*) from the outer to the inner surface of this new generation of dual‐layered e‐PTFE membranes (Trobos, Juhlin, et al., 2018), may lead to a change in the traditional view of e‐PTFE behavior in cases of exposure to the oral environment. This would also open new clinical scenarios for the selection of such barrier, that is, socket preservation, where the e‐PTFE membranes could be left intentionally exposed.

Apart from the influence that the material properties have on bacterial adhesion and biofilm formation, the phenotypic and molecular characteristics of the bacterial species and strains are of importance. In a recent investigation (Harris et al., 2016), CLSM differences in biofilm formation of 98 *Staphylococcus epidermidis* commensal isolates and from various clinical infections (e.g., associated to prosthetic joints and catheters) were classified into five biofilm morphotypes. The results from that study pointed to the fact that the biofilm formation capacity and the structure of the formed biofilm is a complex multifactorial cell–cell adhesion process, which involves complex adaptive genetic mechanisms. Moreover, grouping the isolates into three separate clades showed that the isolates of the disease‐associated clade displayed the most diverse biofilm morphotype (Harris et al., 2016). Interestingly, out of the different biofilm morphology characteristics, only the biofilm thickness revealed a significant relationship with the clade distribution. This suggest that the biofilm thickness confers a strong selective advantage for the bacterial strains that descents from strong biofilm formers.

The ratio between the surface area and the volume of live bacteria was greater, at both time points, on the e‐PTFE membranes. This increased surface area to volume ratio means a more uniform distribution of microorganisms on the substratum, and its clinical significance might be translated into an increased exposure of bacteria to the environment, for example, more access to oxygen and nutrients making cells more metabolically active and responsive than “persister cells” to the action of antibiotics, which in addition could be more available (Davies, 2003). The present findings are in line with data from Trobos, Juhlin, et al., (2018), showing that at different time points, biofilms on d‐PTFE were more mature and thicker (tower formations) than on e‐PTFE, where fewer layers of cells were distributed mainly horizontally along the fiber structures. In another study, three distinct 3D biofilm structural types of *P. aeruginosa* were treated with dispersing agents (Kim, Li, Hwang, & Lee, 2018): The thin flat biofilm was more dislodged than the biofilm with mushroom‐like structure, leaving the thick flat biofilm as the most difficult to break up.

To assess the biofilm cell viability that developed on the barrier membranes, we quantified also the supragingival biofilm present on the buccal surfaces of the lower front teeth of the 12 volunteers. Interestingly, while the number of viable aerobes and anaerobes was greater on the tooth surfaces compared to membranes at both time points, the number of *Staphylococcus* spp. decreased on the tooth surfaces and increased on both membranes over time. Moreover, the number of subjects carrying *Staphylococcus* spp. almost doubled (8/12) when the barrier membranes were introduced in the oral cavity compared to baseline samples (4/12). Staphylococci have long been recognized as benign members of the skin flora, but many species, such as *S. aureus* and *S. epidermidis*, have the capacity to be opportunistic pathogens. Their ability to attach to surfaces and to evade the host defense system leads to the development of refractory biofilm communities that have been linked to a variety of infections, including endocarditis (Parsek & Singh, 2003), osteomyelitis (Brady, Leid, Calhoun, Costerton, & Shirtliff, 2008), abscesses (Cheng, DeDent, Schneewind, & Missiakas, 2011), as well as infections related to medical devices, such as dental implants (Charalampakis, Leonhardt, Rabe, & Dahlen, 2012; Persson & Renvert, 2014), bone‐anchored hearing systems (Trobos, Johansson, et al., 2018), and orthopedic prostheses (Zaborowska et al., 2017).

Our findings support that *S. aureus* could be considered as a frequent isolate of the oral cavity. In patients wearing dentures, *S. aureus* was regularly found both in healthy subjects (Marsh, Percival, & Challacombe, 1992) and in the elderly (Honda, 2001). Furthermore, a 10‐year retrospective analysis of oral and perioral clinical specimens (McCormack et al., 2015), *S. aureus* was detected in 18% of their material samples. The same Figure (18%) was reported at subject level in a Greek population that was either healthy or suffering from periodontal disease (Koukos et al., 2015).

The microbiological finding of *Staphylococcus* spp. on PTFE surfaces in our study agrees with what has been shown in a series of investigations by Merghni and coworkers (Merghni et al., 2016; Merghni et al., 2017; Merghni, Ben Nejma, Hentati, Mahjoub, & Mastouri, 2014). The authors evaluated the adhesive ability of oral staphylococcal strains isolated from the oral cavity and orthodontic appliances or *in vitro* from different abiotic surfaces commonly encountered in dental practices. Interestingly, *S. aureus* exerted the strongest adhesion forces on the PTFE surfaces, whereas the bacterial adhesion force on stainless steel was the lowest. With the complexity of the biofilm communities in the oral cavity, it is not unusual for *Staphylococcus* spp. to be present. These *Staphylococcus* spp. would appear to find some of the biomaterials used to be an ideal microecosystem where they are able to proliferate and, in certain circumstances, may give raise to local or systemic issues. It would therefore be of relevance to consider whether there will be a need to provide local or systemic antimicrobial agents or antiseptics after the manipulation of barrier membranes for GBR procedures to counteract this possible source of infection (Ban et al., 2017; Calon et al., 2019).

The idea of applying devices for intentional *in situ* development and studying of oral biofilm is not new (Prada‐Lopez, Quintas, Vilaboa, Suarez‐Quintanilla, & Tomas, 2016). With respect to GBR membranes, two independent research groups (Simion, Baldoni, Rossi, & Zaffe, 1994; Zucchelli et al., 1998) evaluated the plaque accumulation on various barrier membranes, which were attached to removable acrylic dentures. These devices, built in the laboratory, were adapted to the molar–premolar regions of the upper jaw of dental students. In the present study, the substrate membranes were directly applied on the tooth surfaces and were relatively not voluminous, with the result of being less distressing for esthetics and phonetics.

In the present study, the split‐mouth approach allowed for paired analyses, which may compensate for the relatively small sample size of the study population. Another limitation of the present model is that the possible contact of the PTFE membranes with the muscular action of the cheek and tongue, together with the subsequent removal of the specimens, using small forceps and pliers, might have partially dislodge the biofilms or deflected the appliances. Nevertheless, the differences in the biofilm formed on the two membrane types should still be regarded as representative because the two membrane types were correspondingly placed in each volunteer (spilt‐mouth design). Moreover, an advantage here is that the study also compared some of the characteristics of the membrane‐formed biofilm with the tooth‐formed biofilm (dental plaque).

## 5 CONCLUSION

Results from this study show that the expanded multifibrillar and semiopen structure of a nonresorbable PTFE membrane elicit less biofilm biomass accumulation and biofilm thickness in contrast to solid dense PTFE. Together with previous *in vitro* findings on less bacterial penetration from the outer surface, this findings may lead to a change in the traditional view of e‐PTFE behavior in cases of exposure to the oral environment and would provide new bases for the selection of such barrier for different clinical situations, for example socket preservation where the membrane could be left intentionally exposed. The increased presence of *Staphylococcus* spp. overtime on the two membrane types raises the question for a possible adjustment of antimicrobial strategies, targeting these species, in conjunction with GBR treatment.

## CONFLICT OF INTEREST

All authors declare no conflict of interest.

## Data Availability

The data that support the findings of this study are available on request from the corresponding author. The data are not publicly available due to privacy or ethical restrictions.
